# Utilizing physiologies, transcriptomics, and metabolomics to unravel key genes and metabolites of *Salvia miltiorrhiza* Bge. seedlings in response to drought stress

**DOI:** 10.3389/fpls.2024.1484688

**Published:** 2025-01-14

**Authors:** Yu Zhang, Hong Zhang, Yuru Zhang, Di Wang, Xue Meng, Juan Chen

**Affiliations:** Institute of Chinese Materia Medica, Shaanxi Provincial Academy of Traditional Chinese Medicine, Xi’an, Shaanxi, China

**Keywords:** *Salvia miltiorrhiza* Bunge, drought stress, transcriptomic analysis, metabolomic analysis, AHL gene family

## Abstract

Drought stress inhibits *Salvia miltiorrhiza* Bunge (*S. miltiorrhiza*) seedling growth and yield. Here, we studied the effects of drought stress on the different parts of *S. miltiorrhiza* seedlings through physiological, transcriptomic, and metabolomics analyses, and identified key genes and metabolites related to drought tolerance. Physiological analysis showed that drought stress increased the accumulation of hydrogen peroxide (H_2_O_2_), enhanced the activity of peroxidase (POD), decreased the activity of catalase (CAT) and the contents of chlorophyll b and total chlorophyll, reduced the degree of photosynthesis, enhanced oxidative damage in *S. miltiorrhiza* seedlings, and inhibited the growth of *S. miltiorrhiza* plants. Transcriptome analyses revealed 383 genes encoding transcription factors and 80 genes encoding plant hormones as hypothetical regulators of drought resistance in *S. miltiorrhiza* plants. Moreover, differentially expressed genes (DEGs) and differentially expressed metabolites (DEMs) are involved in a variety of biological processes, such as proline and glycine betaine metabolism, and biosynthesis of tanshinones and phenolic acids. Additionally, it has barely been reported that the AHL gene family may be involved in regulating the neocryptotanshinone biosynthesis. In conclusion, our results suggest that drought stress inhibits *S. miltiorrhiza* seedling growth by enhancing membrane lipid peroxidation, attenuating the antioxidant system, photosynthesis, and regulating proline and glycine betaine metabolism, transcription factors and plant hormones, and tanshinones and phenolic acid metabolism pathways. This study provides new insights into the complex mechanisms by which *S. miltiorrhiza* responds to drought stress.

## Introduction

One of the most challenging natural dangers to measure is a drought environment, which affects soil moisture content, evapotranspiration, atmospheric evaporative demand, and vegetation conditions (Vicente-Serrano et al., 2022). Plants sense the water depletion signal mainly via roots and leaves, and controlling stomatal aperture and closure, which involves some key signaling molecules, including plant hormones, transcription factors (TFs), protein amino acids, reactive oxygen species (ROS), and calcium ion (Ca^2+^) (Liu H et al., 2022; Qiu J et al., 2020; Sharma et al., 2019).

Drought triggers a cascade of physiological and biochemical responses in plants. Photosynthesis is the first mechanism impacted by drought stress, and drought considerably disrupted temperature sensitivities, CO_2_ assimilation rates, gas exchange water-use efficiency, and photosynthetic pigments (Fang et al., 2022; Razi and Muneer, 2021). The antioxidant system is essential for shielding plants from drought stresses via scavenging the ROS overproduced. Peroxidase (POD), superoxide dismutase (SOD), CAT, glutathione *S*-transferase (GST), and aldehyde dehydrogenase (ALDHs) have been found to respond to drought stress (Li R et al., 2022; Han L. et al., 2020). Drought stress renders the maintenance of osmoregulation highly crucial, which depends on the production and buildup of osmolytes including soluble proteins, proline, and sugars (Ozturk et al., 2020; Furlan et al., 2020). TFs are also central regulators of transcriptional reprogramming, and drought stress affected the expression of TF genes and may be involved in metabolite biosynthesis (Thirumalaikumar et al., 2017; Wang et al., 2021; Jadhao et al., 2023). For example, the phenolic acids and anthocyanins were antagonistically regulated by *SmbHLH60* and *SmMYC2* in *Salvia miltiorrhiza*; *SmSPL7* inhibits the production of phenolic acid and facilitates the development of anthocyanins (Liu S et al., 2022; Chen R et al., 2021; Yao et al., 2022; Liu et al., 2021). Moreover, plants can produce hormones to respond to drought stress, which may be closely related to secondary metabolite synthesis (Jafari and Shahsavar, 2021), such as methyl jasmonate (MeJA) stimulating the accumulation of salvianolic acids and tanshinones and triggering the activation of genes in *S. miltiorrhiza* (Pei et al., 2018). Finally, drought stress induces the reduction of yield and quality in plants (Gervais et al., 2021; Han G et al., 2020; Hu et al., 2019). At present, most research focuses on the effects of drought stress on a single system, while the mechanism of multi-system joint resistance to drought stress remains unclear, particularly in the interaction of TFs, plant hormones, and secondary metabolites under drought stress.

*S. miltiorrhiza* is one of the most frequently used herbal remedies with 1,000 years of clinical application in China (Huang et al., 2021). The majority of research on *S. miltiorrhiza* primarily focuses on clinical application, pharmacological activity, and active compounds (Wang et al., 2020; Lu T et al., 2022; MEIm et al., 2019). In northwest China, *S. miltiorrhiza* is extensively cultivated and suffered from the drought environment throughout the year (Chen J et al., 2021). Most importantly, physiological systems, ROS scavenging systems, proline metabolism, TFs, and plant hormones in plants during each development stage are specifically sensitive to drought stress environment (Dietz et al., 2021). The research of drought-stressed *S. miltiorrhiza* focuses on a single system or tissue, lacking comprehensive studies throughout the multi-system and multi-plant tissues (Duan et al., 2023). Therefore, exploring the mechanism of the *S. miltiorrhiza* seedlings’ response to drought stress is crucial for optimizing their growth and yield.

At present, the correlations between MYB, NAC, tanshinones, and phenolic acids in *S. miltiorrhiza* determined through a combination of transcriptomic–metabolomic analysis have been reported (Li S et al., 2022; Yin et al., 2020), but the mechanism of the comprehensive drought resistance is still unclear and incomplete (Zhou et al., 2024). Consequently, in this experiment, to explore the drought response of *S. miltiorrhiza* seedlings, a comprehensive analysis of physiologies, transcriptomics, and metabolomics data was performed. We presumed that (i) drought stress can reduce the degree of photosynthesis and proline and glycine betaine metabolism, enhance oxidative damage, and inhibit the growth in *S. miltiorrhiza* seedlings (Altaf et al., 2022); (ii) the response of TFs and plant hormones to drought stress is closely related (Bhagat et al., 2021); and (iii) there are key gene regulations in the biosynthesis of tanshinones and phenolic acids under drought stress (Zhou et al., 2024). This study provides insight to comprehensively elucidate the molecular mechanism underlying the drought resistance of *S. miltiorrhiza* seedlings. This could serve as a theoretical foundation for further research on the molecular mechanism, breeding, cultivation, and genetic regulation of drought stress resistance in *S. miltiorrhiza*.

## Materials and methods

### Plant materials and treatments

Plants of *S. miltiorrhiza* were grown in a greenhouse under the following conditions: 22°C, light intensity 100 µmol m^−2^ s^−1^, 16/8 h light/dark cycle, and 60% relative humidity. *S. miltiorrhiza* seedlings with essentially the same growth trend were chosen in our study for treatment. A weighing method (saturation moisture content control) was used to simulate water-deficit stress. The experiment was conducted using a randomized design with the following treatments: (i) control group (CK), watering to 75% field capacity; (ii) drought group (CL), water content maintained at 45%. There were 320 seedlings in total and 160 seedlings in each group. After 15 days of treatment, the aerial parts (Y) and underground parts (G) of *S. miltiorrhiza* in each treatment group were collected separately (15 days based on the previous research of the research group) (Chen J et al., 2021). Some samples were used to measure growth and physiological and biochemical characteristics, and the remaining samples were promptly refrigerated at −80°C for transcriptome and metabolome sequencing.

### Measurement of physiological and biochemical characteristics

The contents of chlorophyll, H_2_O_2_, superoxide (O_2_^−^), and proline (Pro), and the activities of SOD, POD, and CAT were determined using reagent kits (Beijing Boxbio Science and Technology Co., Ltd., product numbers 1012311035, 10123122110, 10123112010, 1012311145, 10123112850, 10123112030, and 10123112510). The determination was carried out strictly according to the product instruction.

### Transcriptome analysis

Aerial and underground parts were collected from the same position on the *S. miltiorrhiza* seedlings in each treatment group. The RNA extraction, detection, and cDNA library construction and sequencing were completed by Beijing Nuohezhiyuan Technology Co., Ltd. (Beijing, China). Transcriptome determination was conducted on three biological replicates. The specific methods were as described by Cheng et al. (2023). After the library passed the quality inspection, it was sequenced on an Illumina HiSeq™ 4000 sequencing platform (San Diego, CA, USA). Fragments per kilobase of transcript sequence per millions of base pairs sequenced (FPKM) were used to evaluate the expression level of genes or transcripts. Genes with a fold change > 1 and *p* < 0.05 found via DESeq were determined to be differentially expressed. GO functional enrichment analysis and Kyoto Encyclopedia of Genes and Genomes (KEGG) pathway enrichment analysis were performed using the clusterProfiler 4.2.0 software. Significance threshold for GO functional and KEGG pathways enrichment was both set at *p*_adj_ < 0.05. Gene set enrichment analysis (GSEA) set the threshold values with *p* < 0.05 and FDR < 0.25 using the local version of the GSEA tool (http://www.broadinstitute.org/gsea/index.jsp), and GO and KEGG datasets were used for GSEA independently. As for the correlation analysis of TFs and plant hormones, the differentially expressed genes (DEGs) encoding TFs and plant hormones were imported into the Cytoscape v3.9.1 software.

### Metabolomics analysis

The aerial and underground parts of the *S. miltiorrhiza* seedlings in each treatment group were shipped to Beijing Nuohezhiyuan Technology to conduct qualitative and quantitative analyses of the broad-target metabolome in 24 samples using the LC-QTRAP platform. The specific methods were as described by Cheng et al. (2023). A grinder (MM 400, Retsch, Haan, Germany) was used to crush all tissues to powder (30 Hz, 1.5 min). After being weighed, 100 mg of powder was dissolved in 1.2 mL of 70% methanol. The samples were put at 4°C and vortexed six times, once every 30 min. A microporous membrane with a pore size of 0.22 μm was used to filter the samples after centrifugation (12,000 rpm, 10 min) and the supernatant was aspirated. The samples were then placed in a sample vial for analysis using ultraperformance liquid chromatography–tandem mass spectrometry (UPLC-MS/MS). The secondary spectrum data were used to characterize the metabolites based on a self-constructed database. During the analysis, the repeated and isotopic signals were eliminated. Triple-quadrupole mass spectrometry (TQ-MS) in the multiple reaction monitoring (MRM) mode was used to quantitatively examine the metabolites. Hierarchical cluster analysis (HCA) was used to examine the pattern of metabolite accumulation across the various samples using R software (www.r-project.org). Differentially expressed metabolites (DEMs) were defined as metabolites with VIP > 1 and *p* < 0.05 and fold change ≥ 2 or FC ≤ 0.5, and were annotated, classified, and analyzed by the KEGG database.

### DEG and DEM network analysis

In order to conduct a correlation analysis, the sample sizes must first be consistent. In order to eliminate the impact of magnitude, the data were standardized. The correlation analysis between DEGs and DEMs is based on the Pearson statistical method, calculating the correlation coefficient *r* ^2^ and *p*-value. In the correlation heatmap analysis, the top 50 DEGs and DEMs were determined (sorted by *p*-value from smallest to largest), the top 5 metabolites were selected from the metabolomics enrichment results and the top 10 genes from the transcriptomics, and then the mixOmics package in R software was used to draw a correlation network diagram.

### Statistical analyses

All statistical analyses were performed using GraphPad 9.0 Statistics. Three replications were presented in all treatments; the specific methods of one-way analysis of variance (ANOVA), Duncan’s multiple range tests, and statistical significance were as described by Ming et al. (2023).

## Results

### General description of transcriptome data and metabolome data

The transcriptomic and metabolomic changes in *S. miltiorrhiza* seedlings under different groups were compared. Detailed statements of transcriptome data are presented in **Supplementary Table S1**. The 8,588 and 6,327 DEGs were screened in G_CL vs. G_CK groups (3,657 upregulated and 4,931 downregulated), and Y_CL vs. Y_CK groups (2,699 upregulated and 3,628 downregulated), respectively (**Supplementary Figure S1A**). The heatmap visualized distinct hierarchical clustering of genes, suggesting that these genes have different expression patterns in response to the drought stress (**Supplementary Figure S1B**). A heatmap of DEMs was constructed in two-ion mode, which clearly reflects the different impacts of drought stress on the aerial and underground parts of *S. miltiorrhiza* seedlings (**Supplementary Figures S1C, D**). Moreover, we identified 80 common DEMs between the G_CL vs. G_CK and Y_CL vs. Y_CK groups in positive ion mode, and 34 in negative ion mode (**Supplementary Figures S1E, F**).

### Plant growth parameters and photosynthesis

**Figure 1:**
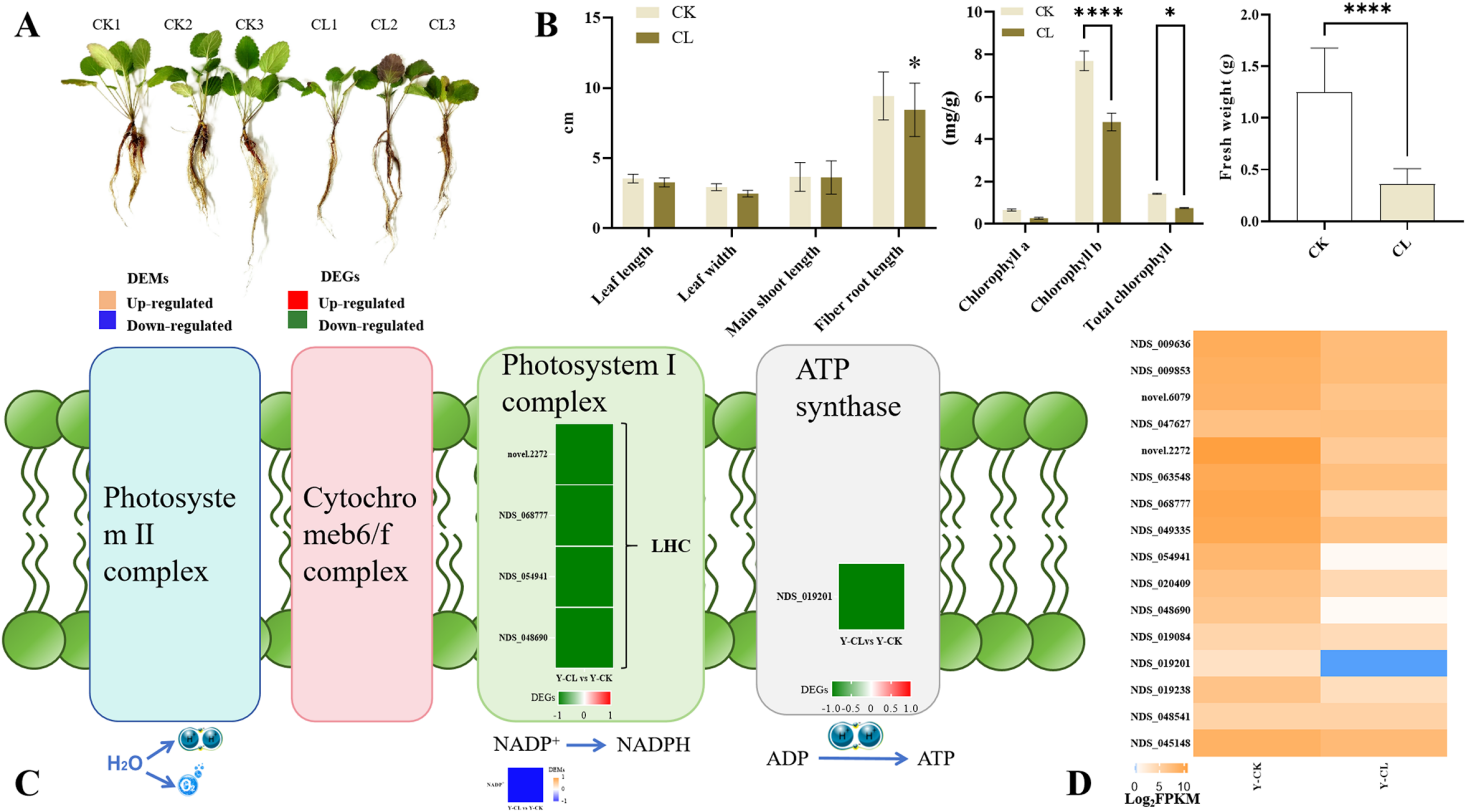
Effects of drought stress on growth and photosynthesis in *S.miltiorrhiza* seedling. **(A)** Growth situation in different conditions in *S.miltiorrhiza* seedlings. **(B)** Growth index and photosynthesis index in different conditions. **(C)** Expression patterns of the differentially expressed genes (DEGs) and differentially expressed metabolites (DEMs) related to photosynthesis. **(D)** Expression patterns of the genes related to photosynthesis. Values are the mean ± SE(n=3). Genes with *p*<0.05 and fold change≥1.5 were considered differentially expressed, metabolites with *p*<0.05, and variable importance in projection (VIP)>1, and fold change≥1 were considered differentially expressed. The “ * “ stands for the significant difference at *p*<0.05. The “ **** “ stands for the significant difference at *p*<0.001.

The change of morphological characteristics in stressed *S. miltiorrhiza* seedlings was performed (**Figure 1A**). Relative to the control group, drought stress inhibited the growth of *S. miltiorrhiza* seedlings by decreasing fiber root length, chlorophyll b, total chlorophyll, and fresh weight, but chlorophyll a did not show significant changes (**Figure 1B**). The four genes encoding *LHC* (photosystem I complex) and one gene encoding *ATP synthase* were downregulated in the Y-CL vs. Y-CK comparison (**Figures 1C, D**). Moreover, metabolomic analysis showed that the content of NADP^+^ decreased in the Y-CL vs. Y-CK comparison (**Figure 1C**).

### ROS scavenging process

**Figure 2:**
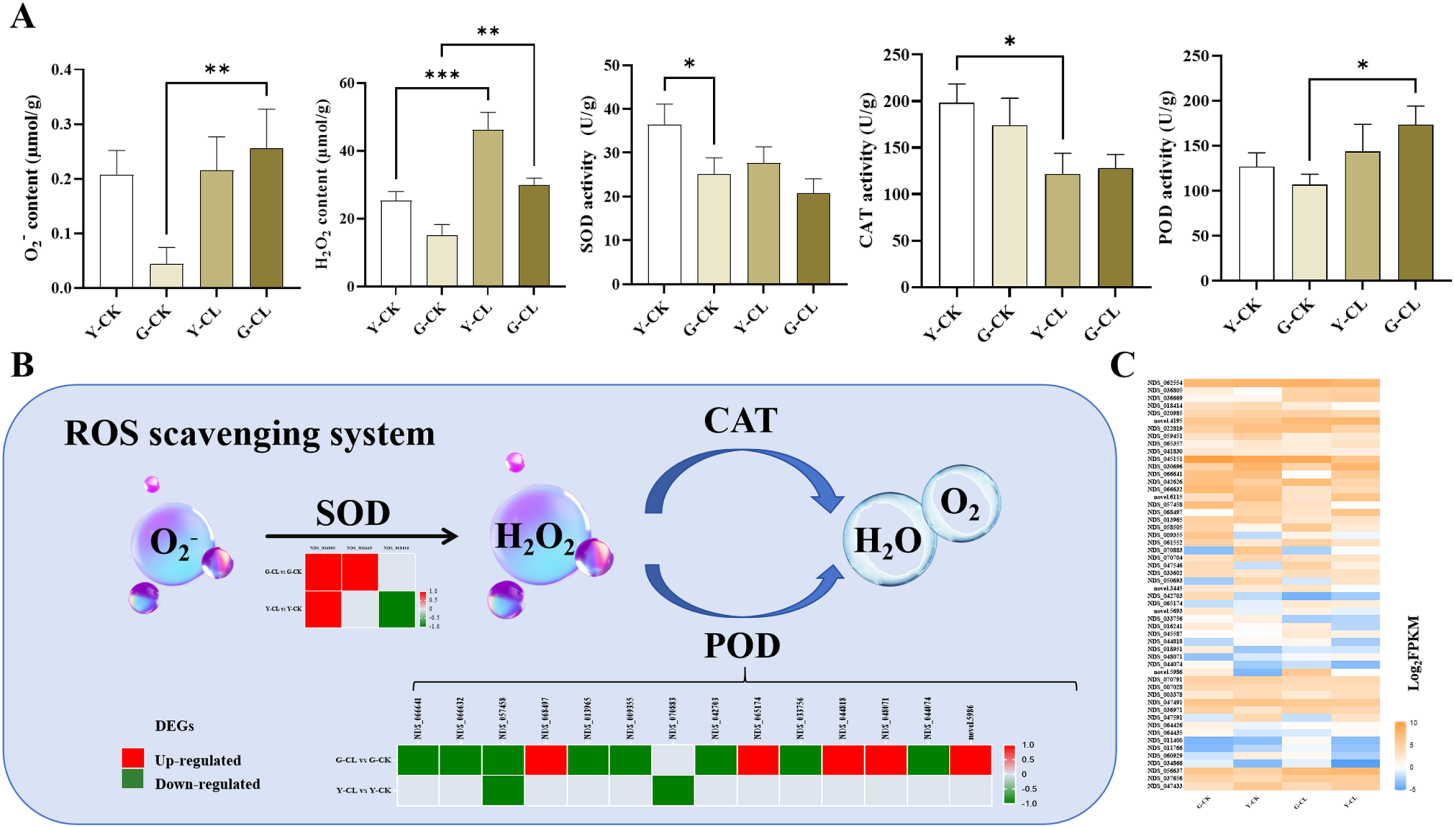
Effects of drought stress on ROS scavenging process in *S.miltiorrhiza* seedling. **(A)** the contents of ROS scavenging process markers and activities of the antioxidant enzymes. **(B)** Expression patterns of the DEGs related to antioxidant enzymes. **(C)** Expression patterns of the genes related to ROS scavenging process. Values are the mean ± SE(n=3). Genes with *p*<0.05 and fold change≥1.5 were considered differentially expressed. The “ * “ stands for the significant difference at *p*<0.05. The “ ** “ stands for the significant difference at *p*<0.01. The “ *** “ stands for the significant difference at *p*<0.005.

The contents of O_2_^−^ and H_2_O_2_ significantly increased in the G-CL vs. G-CK comparison, and the content of H_2_O_2_ also significantly increased in the Y-CL vs. Y-CK comparison (**Figure 2A**). The activity of CAT significantly decreased in the Y-CL vs. Y-CK comparison; however, the activity of POD significantly increased in the G-CL vs. G-CK comparison (**Figure 2A**). The genes of different groups encoding the ROS scavenging process were differentially expressed (**Figures 2B, C**). Two genes encoding *SOD* and five genes encoding *POD* were upregulated; inversely, eight genes encoding *POD* were downregulated in the G-CL vs. G-CK comparison and 1 gene encoding *SOD* was upregulated; in contrast, one gene encoding *SOD* and two genes encoding *POD* were downregulated in the Y-CL vs. Y-CK comparison (**Figure 2B**).

### Proline and glycine betaine metabolism

**Figure 3:**
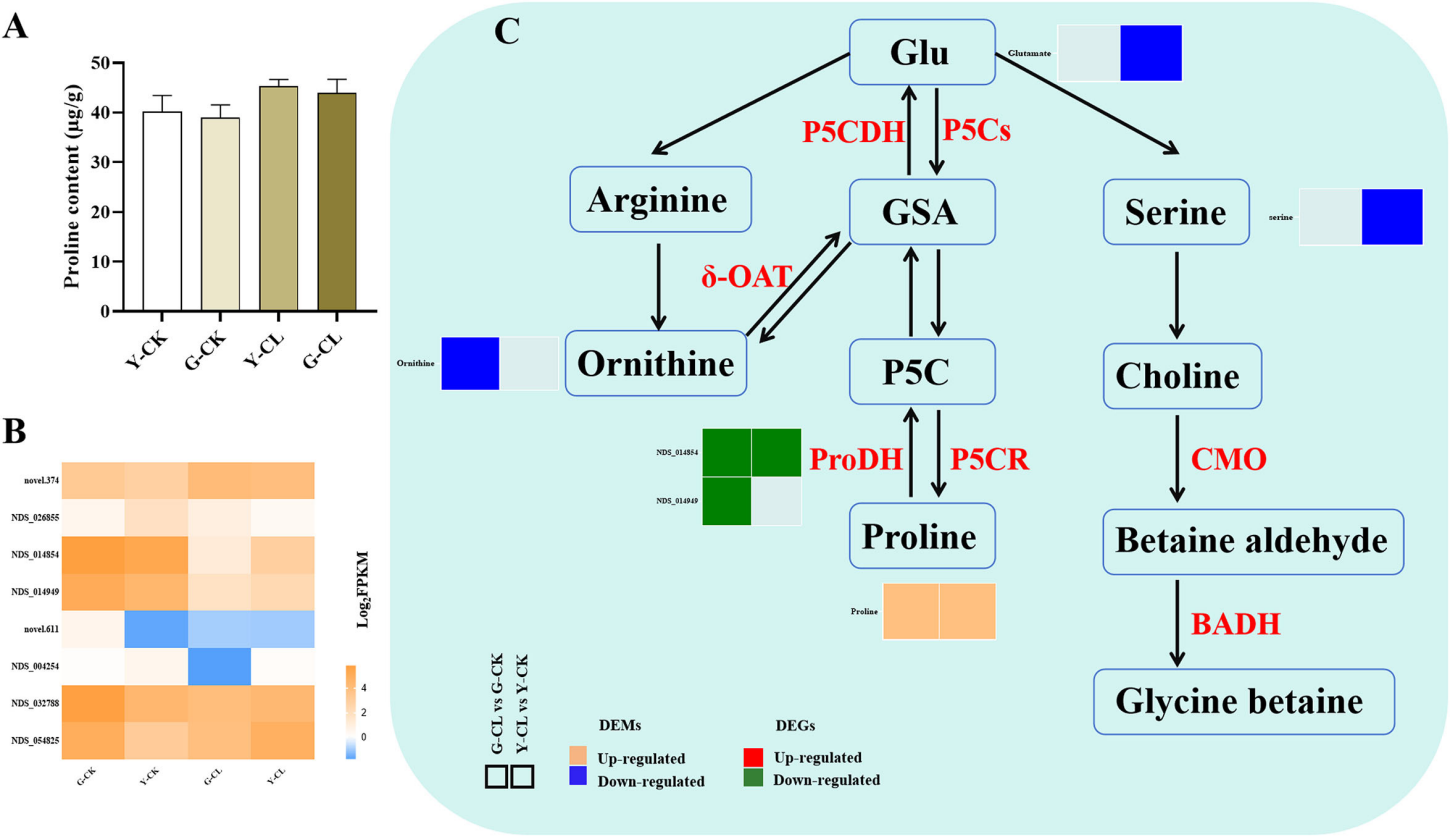
Effects of drought stress on proline and glycine betaine metabolism in *S.miltiorrhiza* seedling. **(A)** the content of proline. **(B)** Expression patterns of the genes related to proline and glycine betaine metabolism. **(C)** Expression patterns of the DEGs and DEMs related to proline and glycine betaine metabolism. Values are the mean ± SE (n=3). Genes with p<0.05 and fold change≥1.5 were considered differentially expressed, metabolites with *p*<0.05, and variable importance in projection (VIP)>1, and fold change≥1 were considered differentially expressed.

Physiological data were combined with transcriptomic and metabolomic data to obtain a detailed pathway for proline and glycine betaine metabolism (**Figure 3**). The two genes encoding *ProDH* were downregulated in the G-CL vs. G-CK comparison, and one gene encoding *ProDH* was downregulated in the Y-CL vs. Y-CK comparison (**Figure 3C**). Moreover, the metabolomic analysis showed that the content of ornithine decreased in the G-CL vs. G-CK comparison, and the contents of glutamate (Glu) and serine decreased in the Y-CL vs. Y-CK comparison (**Figure 3C**). Inversely, the content of proline increased in both comparisons (**Figure 3C**).

### Transcription factors and plant hormones

**Figure 4:**
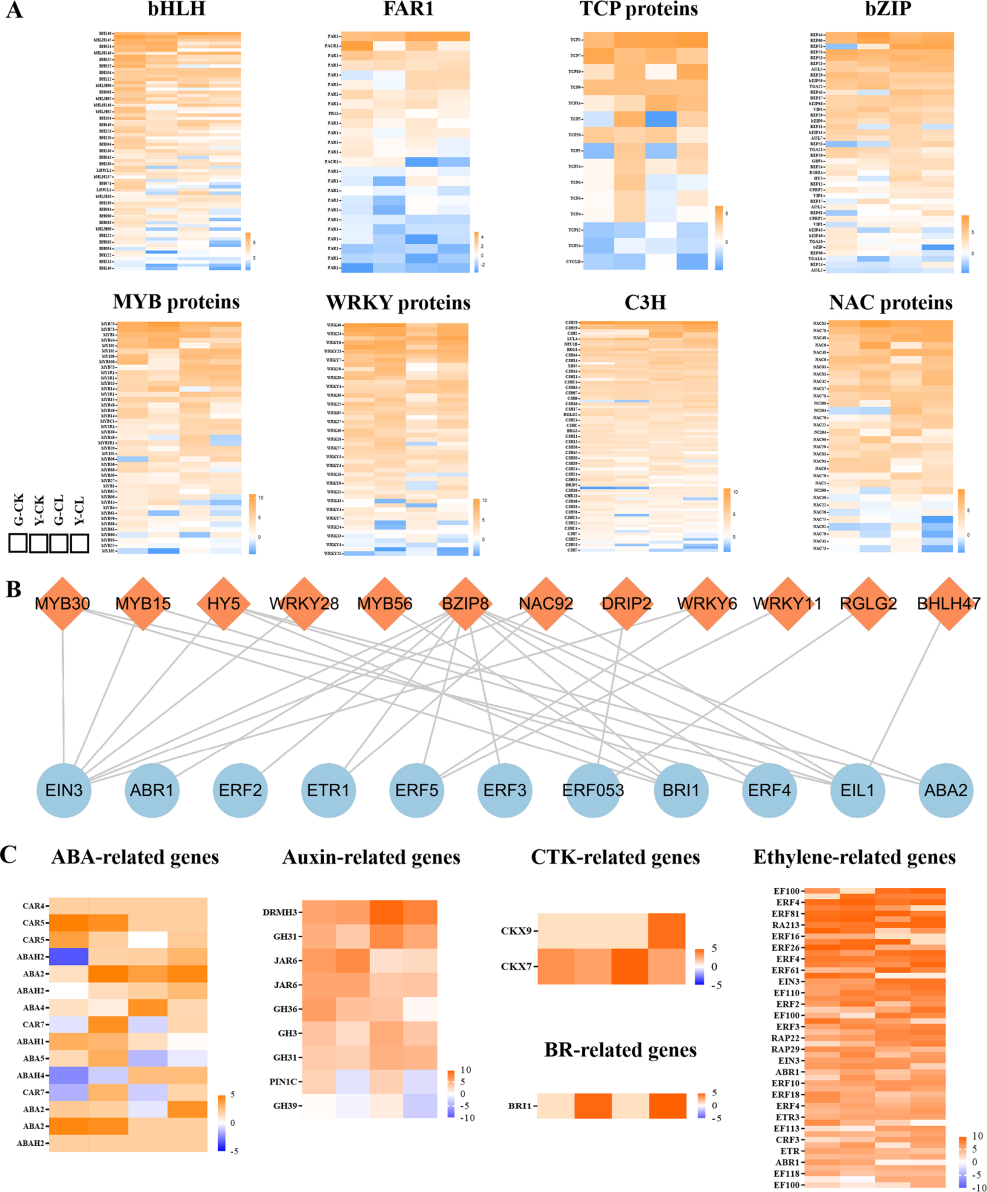
Effects of drought stress on transcription factors and plant hormones in *S.miltiorrhiza* seedling. **(A)** Expression patterns of the genes related to transcription factors. **(B)** the correlation of transcription factors and plant hormones. **(C)** Expression patterns of the genes related to plant hormones.

TFs and plant hormones play an important regulatory role in plant responses to drought stress. In this study, a total of 383 TFs and 80 plant hormones were identified as hypothetical regulators of drought resistance in *S. miltiorrhiza* (**Figure 4**). TFs include 73 genes encoding *basic helix-loop-helix* (*bHLHs*), 25 genes encoding *far-red impaired response 1* (*FAR1s*), 15 genes encoding *TCP* proteins (*TCPs*), 42 genes encoding *basic region-leucine zipper* (*bZIPs*), 43 genes encoding *MYB* domain proteins (*MYBs*), 53 genes encoding *WRKY* proteins (*WRKYs*), 85 genes encoding *Cys3His* zinc finger proteins (*C3Hs*), and 32 genes encoding *NAC* proteins (*NACs*) (**Figure 4A**). Plant hormones include 15 genes encoding abscisic acid (ABA), 9 genes encoding auxin, 2 genes encoding cytokinin (CTK), 1 gene encoding brassinolide (BR), and 53 genes encoding ethylene (**Figure 4C**).

Moreover, the correlation of TFs and plant hormones was analyzed (**Figure 4B**). The genes encoding ethylene were correlated with genes encoding *bZIP*, *bHLH*, *C3H*, *NAC*, *MYB*, and *WRKY*. Genes encoding BR were correlated with genes encoding *MYB* and *bZIP*. In addition, genes encoding ABA were correlated with genes encoding *bZIP*.

### Biosynthesis of tanshinones and phenolic acids

**Figure 5:**
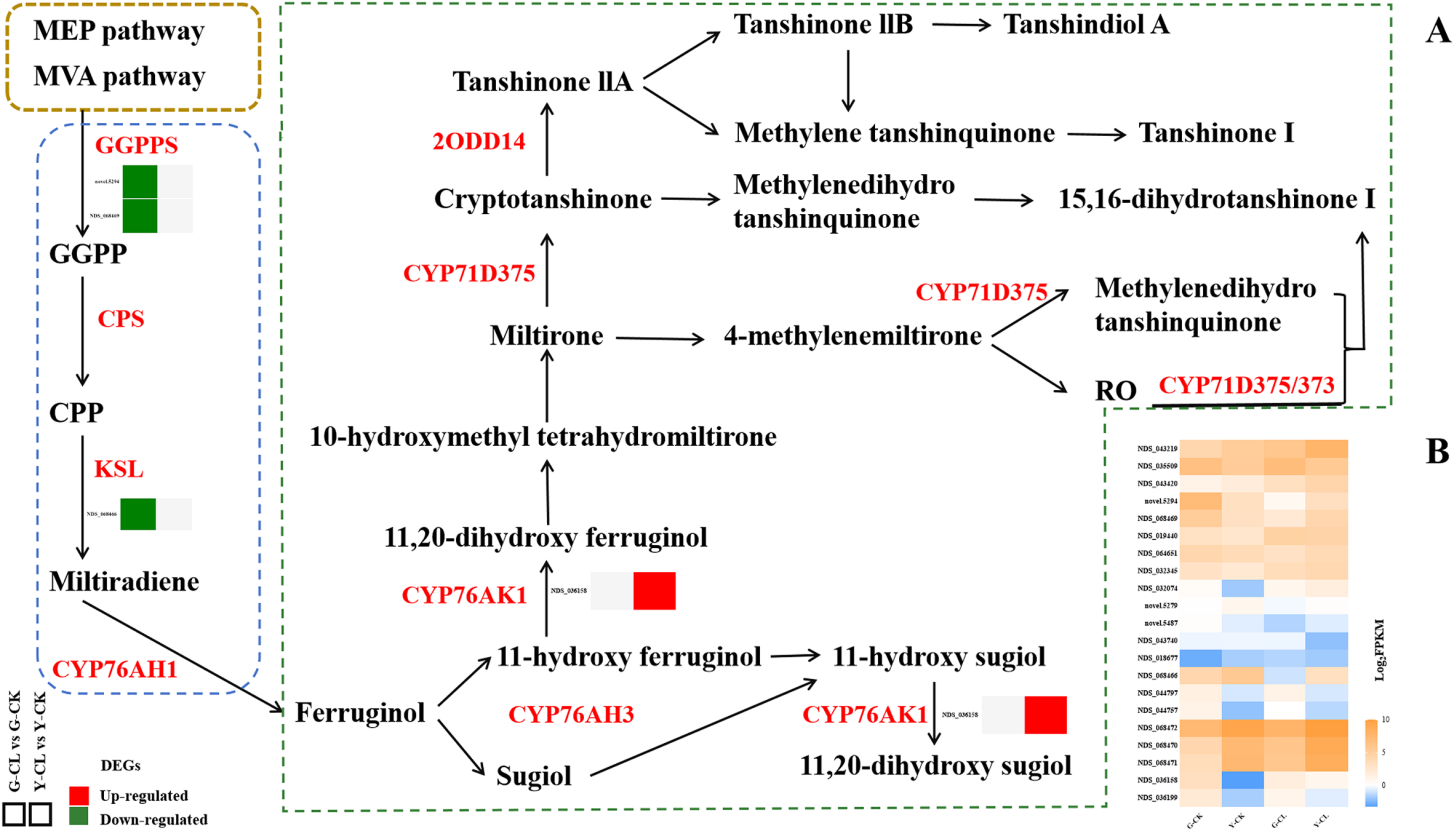
Effects of drought stress on tanshinones biosynthesis. **(A)** Expression patterns of the DEGs related to tanshinones biosynthesis. **(B)** Expression patterns of the genes related to tanshinones biosynthesis. Genes with *p*<0.05 and fold change≥1.5 were considered differentially expressed.

**Figure 5** displays the tanshinones biosynthesis pathway. Transcriptomic analysis showed that the two genes encoding *GGPPS* and one gene encoding *KSL* were downregulated in the G-CL vs. G-CK comparison, respectively, and one gene encoding *CYP76AK1* was upregulated in the Y-CL vs. Y-CK comparison.

**Figure 6:**
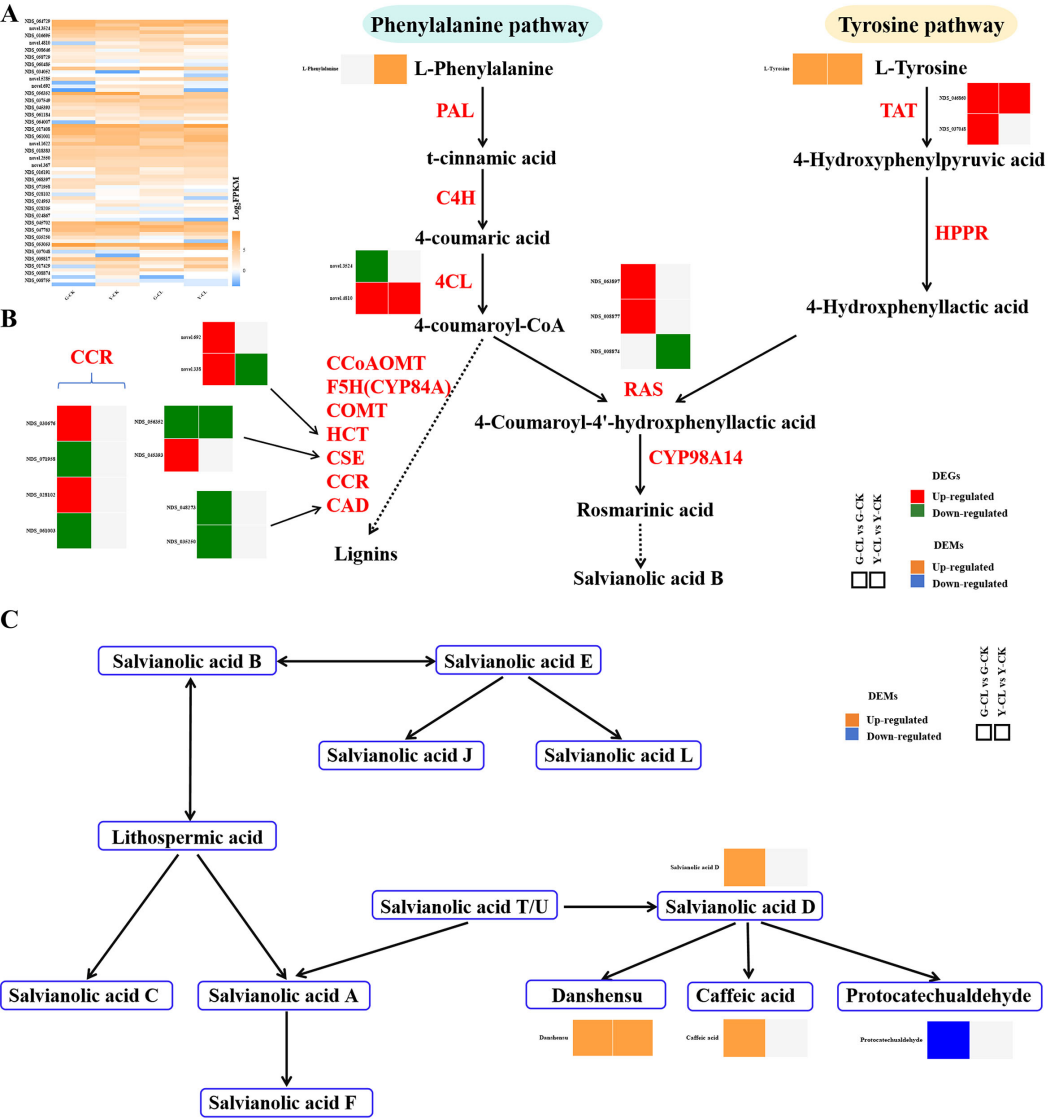
Effects of drought stress on phenolic acids biosynthesis. **(A)** Expression patterns of the genes related to phenolic acids biosynthesis. **(B, C)** Expression patterns of the DEGs and DEMs related to phenolic acids biosynthesis. Genes with *p*<0.05 and fold change≥1.5 were considered differentially expressed, metabolites with *p*<0.05, and variable importance in projection (VIP)>1, and fold change≥1 were considered differentially expressed.

**Figure 6** displays salvianolic acid B (**Figures 6A, B**) and phenolic acid (**Figure 6C**) pathways. Transcriptomic analysis showed that one gene encoding *4CL*, one gene encoding *CSE*, two genes encoding *CCR*, and two genes encoding *CAD* were downregulated, but one gene encoding *4CL*, two genes encoding *HCT*, one gene encoding *CSE*, two genes encoding *CCR*, two genes encoding *RAS*, and two genes encoding *TAT* were upregulated in the G-CL vs. G-CK comparison (**Figure 6B**). In the Y-CL vs. Y-CK comparison, one gene encoding *HCT*, one gene encoding *CSE*, and one gene encoding *RAS* were downregulated; inversely, one gene encoding *4CL* and one gene encoding *TAT* were upregulated (**Figure 6B**). Moreover, the metabolomic analysis showed that the content of L-tyrosine, salvianolic acid D, danshensu, and caffeic acid increased, but the content of protocatechualdehyde decreased in the G-CL vs. G-CK comparison; the content of L-phenylalanine, L-tyrosine, and danshensu increased in the Y-CL vs. Y-CK comparison (**Figures 6B, C**).

### The transcriptome and metabolome network analysis

**Figure 7:**
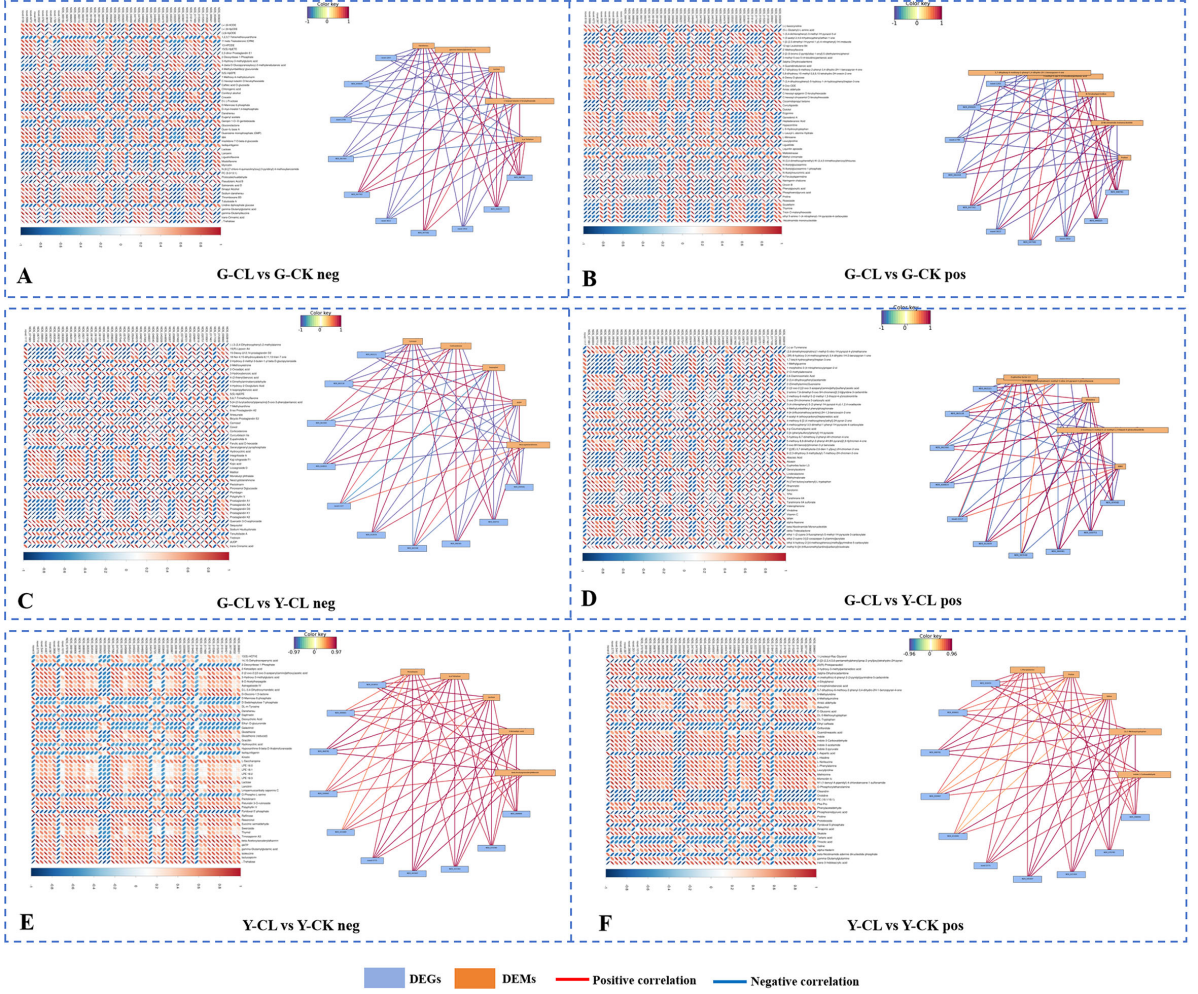
Association analysis of DEGs and DEMs. Heatmap of the expression of Top 50 DEGs (horizontal) and Top 50 DEMs (vertical) that differ in positive ion mode (positive) and negative ion mode (negative). **(A, B)** G-CL vs G-CK in negative and positive mode. **(C, D)** G-CL vs Y-CL in negative and positive mode. **(E, F)** Y-CL vs Y-CK in negative and positive mode. Network of the expression of Top 10 DEGs (blue) and Top 5 DEMs (yellow) that differ in positive ion mode (positive) and negative ion mode (negative). The positive correlation is red, and negative correlation is blue. Genes with p<0.05 and fold change≥1.5 were considered differentially expressed, metabolites with p<0.05, and variable importance in projection (VIP)>1, and fold change≥1 were considered differentially expressed. The p-values are ordered from smallest to largest.

In the G-CL vs. G-CK comparison (negative ion mode) (**Figure 7A**), danshensu was negatively correlated with 18 genes and positively correlated with 28 genes, with 1 gene encoding *CYP450* (*novel.1263*) negatively correlated and 1 gene encoding *PILS2* (*NDS_047582*) and 1 gene encoding *MLO6* (*NDS_068781*) positively correlated; salvianolic acid D was negatively correlated with 18 genes and positively correlated with 30 genes. In the G-CL vs. G-CK comparison (positive ion mode) (**Figure 7B**), proline was negatively correlated with 18 genes and positively correlated with 29 genes, with 1 gene encoding *CYP450* (*novel.1263*) negatively correlated and 1 gene encoding *PILS2* (*NDS_047582*) and 1 gene encoding *MLO6* (*NDS_068781*) positively correlated.

In the G-CL vs. Y-CL comparison (negative ion mode) (**Figure 7C**), carnosol was negatively correlated with two genes encoding *NLTP2* (*NDS_062121*; *NDS_062118*), one gene encoding *Y1457* (*NDS_020819*), one gene encoding *CB4A* (*NDS_063548*), and one gene encoding *GEDH1* (*NDS_059582*); inversely, carnosol was positively correlated with one gene encoding *AAP3* (*NDS_012939*), one gene encoding *AHL20* (*NDS_060381*), and one gene encoding *PTR6Z* (*NDS_020751*). Moreover, the correlation of neocryptotanshinone (NCTS) was the same as that of carnosol. In the G-CL vs. Y-CL comparison (positive ion mode) (**Figure 7D**), ABA was negatively correlated with 24 genes and positively correlated with 15 genes, and tanshinone IIA was negatively correlated with 24 genes and positively correlated with 21 genes.

In the Y-CL vs. Y-CK comparison (negative ion mode) (**Figure 7E**), danshensu was negatively correlated with 2 genes and positively correlated with 22 genes. α,α-trehalose was positively correlated with one gene encoding *ADH1* (*NDS_019455*), one gene encoding *EXLB1* (*NDS_006601*), one gene encoding *MLO6* (*NDS_068781*), two genes encoding *UGT2* (*NDS_030967* and *NDS_031009*), one gene encoding *amino acid permease 7* (*novel.5775*), one gene encoding *TDT* (*NDS_045007*), one gene encoding *HSP7C* (*NDS_015364*), one gene encoding *EDL3* (*NDS_070785*), and one gene encoding *SKU5* (*NDS_068906*). In the Y-CL vs. Y-CK comparison (positive ion mode) (**Figure 7F**), proline was negatively correlated with 5 genes and positively correlated with 15 genes. In particular, the correlation results of proline and valine were the same as that of α,α-trehalose.

### GO and KEGG enrichment analysis

The GO terms “oxidoreductase activity” and “transcription regulator activity” were highly enriched among gene sets in the G_CL vs. G_CK group, and the GO terms “photosystem”, “photosynthetic membrane”, and “thylakoid part” were highly enriched among gene sets in Y_CL vs. Y_CK (**Supplementary Figures S2A, B**). The KEGG terms “Plant hormone signal transduction”, “MAPK signaling pathway - plant”, and “Peroxisome” were the significantly enriched different genes in the G_CL vs. G_CK group, and the KEGG terms “Plant hormone signal transduction”, “MAPK signaling pathway-plant”, “Photosynthesis”, and “Photosynthesis - antenna proteins” were the significantly enriched different genes in Y_CL vs. Y_CK (**Supplementary Figures S2C, D**).

### Gene set enrichment analysis

**Figure 8:**
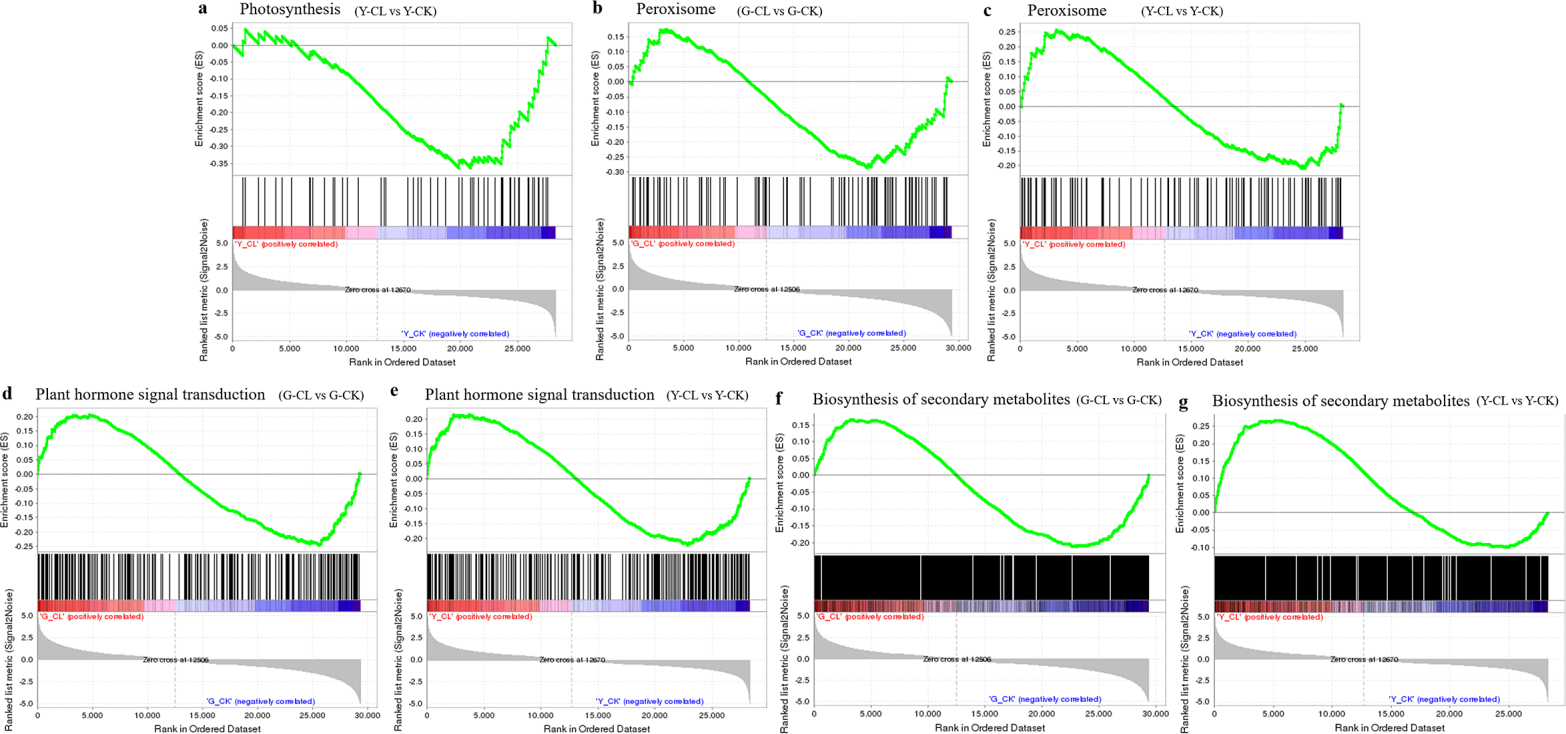
GSEA analysis of photosynthesis **(A)**, peroxisome **(B, C)**, plant hormone signal transduction **(D, E)**, and biosynthesis of secondary metabolites pathway **(F, G)** in different treatments with p<0.05, FDR <0.25.

GSEAs of the photosynthesis pathway (ATH00195), peroxisome pathway (ATH04146), MAPK signaling pathway (ATH04016), plant hormone signal transduction pathway (ATH04075), and biosynthesis of secondary metabolites pathway (ATH01110) were performed to ascertain the function changes in response to drought stress in *S. miltiorrhiza* seedlings (**Figure 8**). In the Y-CL vs. Y-CK comparison, the photosynthesis pathway was significantly enriched and downregulated (**Figure 8A**). However, peroxisome, MAPK signaling pathway, plant hormone signal transduction, and biosynthesis of secondary metabolites pathways were overall upregulated in two comparisons (**Figures 8B-G**).

## Discussion

### Drought stress affects growth indexes and photosynthesis of *S. miltiorrhiza* seedlings

During plant cultivation, drought stress affects morphological and physiological changes in plants, which has a great impact on its growth and productivity (Lim et al., 2022). In this research, drought stress decreased fiber root length and fresh weight in *S. miltiorrhiza* seedlings (**Figure 1B**), so we predicted that root morphology would be firstly negatively affected under drought stress (Verbraeken et al., 2021; Gervais et al., 2021; Altaf et al., 2022). Moreover, drought stress decreased the contents of chlorophyll b, total chlorophyll, and NADP^+^, and downregulated the genes of encoding *LHC* and *ATP synthase* (**Figures 1B, C**), which were similar to the results of the GO, KEGG, and GSEA (**Figure 8A**; **Supplementary Figures S2B, D**). PSI and the ATP synthase are embedded where solar energy is collected and converted into NADPH and ATP (Jonwal et al., 2022). LHC proteins collect light energy and photoprotection and regulate chlorophyll synthesis in plants (Rochaix and Bassi, 2019). Therefore, in our research, drought stress inhibited the expression of genes encoding *LHC* and *NADP^+^* supply, reduced light harvest, and thereby decreased the content of chlorophyll, which slowed down the growth of *S. miltiorrhiza* seedlings (Zhang et al., 2022).

### Drought stress affects the ROS scavenging process of *S. miltiorrhiza* seedlings

Plants benefit from the ROS scavenging mechanism during drought stress, because it allows them to modify their metabolism and mount an appropriate acclimation response (Gao et al., 2022). Our KEGG and GSEA results showed that the peroxisome pathway was significantly enriched and upregulated (**Figures 8B, C**; **Supplementary Figure S2C**), which is consistent with the changes of SOD and POD (**Figures 2A, B**), and may be involved in ROS detoxification by accelerating the dismutation of O_2_^−^ into H_2_O_2_ and scavenging the H_2_O_2_ under drought stress mainly by the increase in PODs rather than CATs (Ritonga et al., 2021; Balfagón et al., 2021; Hou et al., 2021). Interestingly, the results of transcriptomic analysis and enzyme activity on POD in the G-CL vs. G-CK comparison were not consistent (**Figures 2A, B**), indicating that these genes might not be key genes for the regulation of POD activity.

### Drought stress affects the proline and glycine betaine metabolism of *S. miltiorrhiza* seedlings

Drought stress renders the maintenance of osmoregulation highly vital (Tan et al., 2022). Specifically, biosynthesis of proline and glycine betaine in plants occurs through Glu pathways, catalyzed by three pathways (**Figure 3C**). In our study, drought stress increased the content of proline and downregulated the expression of genes encoding *ProDH* (**Figure 3C**); the result might be due to drought stress mainly generating pyrroline-5-carboxylate (P5C) and then further reduced by P5CR to generate proline, rather than proline degradation pathway by ProDH (Ingrisano et al., 2023). Moreover, the content of ornithine and serine decreased due to drought stress (**Figure 3C**), and it was further proved that drought stress mainly provided sufficient substrate for proline by GSA, and the decrease in the ornithine and serine content might be due to one part being used to synthesize proline (Shafiq et al., 2021). Interestingly, we found in the G-CL vs. G-CK comparison (positive ion mode) (**Figure 7B**) that proline was positively correlated with one gene encoding *PILS2* (*NDS_047582*). PILS can give a homeostatic feedback on the auxin signaling pathway, and proline and auxin signal transduction pathways are also crucial mechanisms for coping with drought stress, probably indicating that proline and auxin combined to respond to drought stress in *S. miltiorrhiza* seedlings (Feraru et al., 2022).

### Drought stress affects the transcription factors and plant hormones of *S. miltiorrhiza* seedlings

Stress-responsive genes are expressed due to TFs, which are a crucial component of the adaptive stress process (Lu X et al., 2022; Ju et al., 2019; Joo et al., 2021; Han G et al., 2020; Xuan et al., 2022). In our study, bHLHs, WRKYs, and C3Hs are more sensitive to drought stress in *S. miltiorrhiza* seedlings (**Figure 4A**), because of which bHLHs, WRKYs, and C3Hs could regulate cellular activities and membrane-lipid stability, increase drought tolerance, and promote root growth and water retention (Qiu X. et al., 2020; Xiang et al., 2021). In addition, plant hormone homeostasis is critical to regulating plant growth and development under stress conditions, and multiple levels are regulated (Zhang et al., 2023; Wang and Qiao, 2020; Li et al., 2020; Waadt et al., 2022). In our study, drought stress regulated expression genes of ethylenes, ABAs, auxins, CTKs, and BRs, of which ABAs and ethylenes were encoded the most (**Figure 4C**), which was consistent with KEGG and GSEA results (**Figures 8D, E**; **Supplementary Figure S2C**). ABA and ethylene play key roles in mediating vessel size, cell wall thickness, water loss, and oxidative damage in plant responses to drought stress (Gambhir et al., 2022; Auler et al., 2020; Kong et al., 2023).

Interestingly, the correlation results of TFs and plant hormones showed that *NAC92* was correlated with *EIN3*, *ETR1*, and *EIL1* (**Figure 4B**), which were involved in regulating chlorophyll metabolism, the carotenoid biosynthetic process, and ethylene pathways (He et al., 2022; Luo et al., 2019). We found that *EIN3* and *EIL1* can bind to several gene members of *MYB* and *bHLH* (**Figure 4B**), which may mainly the regulate ethylene signaling pathway, thereby promoting root hair formation (Wen et al., 2023; Song et al., 2022). Moreover, we found that *bZIP8* may be a key gene, because it involves most genes encoding plant hormones (**Figure 4B**), but little was known. Currently, it is known that *BRI1* and *ABAs* are related to *bZIP8* and regulate cell communication, membrane signaling, plant development, photomorphogenesis, flavonoid biosynthesis, nutrient acquisition, and response to drought stress (Lozano-Elena and Caño-Delgado, 2019; Bhagat et al., 2021; Xiao et al., 2022; Nie et al., 2022). According to these reports and our study results, the combination of key TFs and plant hormones might play a specific role in drought-stressed *S. miltiorrhiza* seedlings. These key genes are involved in regulating seedling growth, photosynthesis, the ROS scavenging system, proline metabolism, etc., and they are worthy of further research and development.

### Drought stress affects the biosynthesis of tanshinones and phenolic acids of *S. miltiorrhiza* seedlings

Tanshinones (liposoluble) and phenolic acids (water-soluble) are a series of crucial compounds in *S. miltiorrhiza* (Shi et al., 2022). In our study, we only found that drought stress downregulated some genes encoding *GGPPS* and *KSL* in the tanshinones biosynthesis pathway (**Figure 5**); this result indicates that the effect of the tanshinones biosynthesis pathway in the present seedlings growth stage is relatively minimal, or the effect of drought stress on the tanshinones biosynthesis pathway is not significant (Loyola et al., 2011). Surprisingly, in the G-CL vs. Y-CL comparison (negative ion mode) (**Figure 7C**), we found that NCTS was correlated with genes encoding *NLTP2*, *Y1457*, *CB4A*, *GEDH1*, *AAP3*, *AHL20*, and *PTR6Z*, which related to the salicylic acid biosynthesis pathway and nutrient transport (Silverio-Gómez et al., 2021; Zhang et al., 2021). At present, the effect of drought stress on NCTS and AHL gene family has not been reported in *S. miltiorrhiza* seedlings (Ma et al., 2023). Studies have shown that NCTS is a diterpenoid with a similar structure to these tanshinones, and it has anti-inflammatory and blood-activating effects (Yang et al., 2022). AHL family members play a critical role in stress resistance regulation by DNA and protein interactions in plant biological processes (Zhao et al., 2020; Kumar et al., 2023). Therefore, the finding of NCTS and AHL gene family can provide more evidence and potential on the tanshinones pathway and growth and development in *S. miltiorrhiza*.

The phenolic acids biosynthesis pathway was significantly regulated under drought stress (**Figure 6**). Interestingly, in the present study, drought stress was significantly involved in lignin synthesis, indicating that lignin synthesis was sensitive to drought stress and lignin deposition in the plant secondary cell wall as a protection to drought attacks (Sharma et al., 2023). Moreover, genes encoding *RAS* upregulated in the G-CL vs. G-CK comparison (**Figure 6B**), which might accelerate the synthesis of salvianolic acid B in *S. miltiorrhiza* and upregulate the genes’ expression encoding *TAT* in the tyrosine pathway (Zhong et al., 2023; Wagay et al., 2023). In our study, the contents of salvianolic acid D, danshensu, and caffeic acid increased in the G-CL vs. G-CK comparison (**Figure 6C**), indicating that correlations were observed between drought stress and salvianolic acid accumulation, and this chemical is also the most responsive to drought stress. Above all, the secondary metabolite pathway was significantly enriched in two comparisons and was upregulated (**Figures 8F, G**). Interestingly, danshensu was negatively correlated with one gene encoding *CYP450* (*novel.1263*) in the G-CL vs. G-CK comparison (negative ion mode) (**Figure 7A**), but CYPs played a continuous catalytic role in the tanshinones biosynthesis pathway (Mao et al., 2020); therefore, this gene could redirect both salvianolic acids and tanshinones biosynthesis possibly through bidirectional regulation. Danshensu was positively correlated with one gene encoding *PILS2* (*NDS_047582*) and one gene encoding *MLO6* (*NDS_068781*) (**Figure 7A**), which indicates that drought stress may produce the change of network system related to gene *PILS*, auxin, and danshensu (Bogaert et al., 2021; Li Q. et al., 2022). In addition, we also found that salvianolic acid D was negatively correlated with 18 genes and positively correlated with 30 genes (**Figure 7A**). However, at present, there are only a few reports on these genes and phenolic acids in plants; their response to drought stress in *S. miltiorrhiza* seedlings needs to be clarified in future research.

## Conclusion

This study found that *S*. *miltiorrhiza* seedlings can quickly regulate gene expression, enzyme activity, and metabolism content, which is involved in photosynthesis, the ROS scavenging process, proline and glycine betaine metabolism, TFs, plant hormones, biosynthesis of tanshinones and phenolic acids, and adapting to drought stress conditions. We found that *bZIP8* may be a key gene as it involves most genes encoding plant hormones, which regulate cell communication, membrane signaling, plant development, and response to drought stress. The relationship between NCTS and the AHL gene family plays pivotal roles in *S*. *miltiorrhiza*’s response to drought stress at the tanshinones pathway and various growth stages. These findings substantially enhance our understanding of the mechanisms underlying *S*. *miltiorrhiza*’s response to drought stress and provide new insights into the functions of relevant genes and metabolites in drought tolerance.

## Data Availability

The datasets presented in this study can be found in online repositories. The names of the repository/repositories and accession number(s) can be found in the article/**Supplementary Material**.
