# Supplementary material for: Utilizing physiologies, transcriptomics, and metabolomics to unravel key genes and metabolites of Salvia miltiorrhiza Bge. seedlings in response to drought stress
Source: Front Plant Sci. 2025 Jan 14;15:1484688. doi: 10.3389/fpls.2024.1484688 (PMC11772496; doi:10.3389/fpls.2024.1484688)
Supplement: Supplementary file 1 [file Table1.doc]

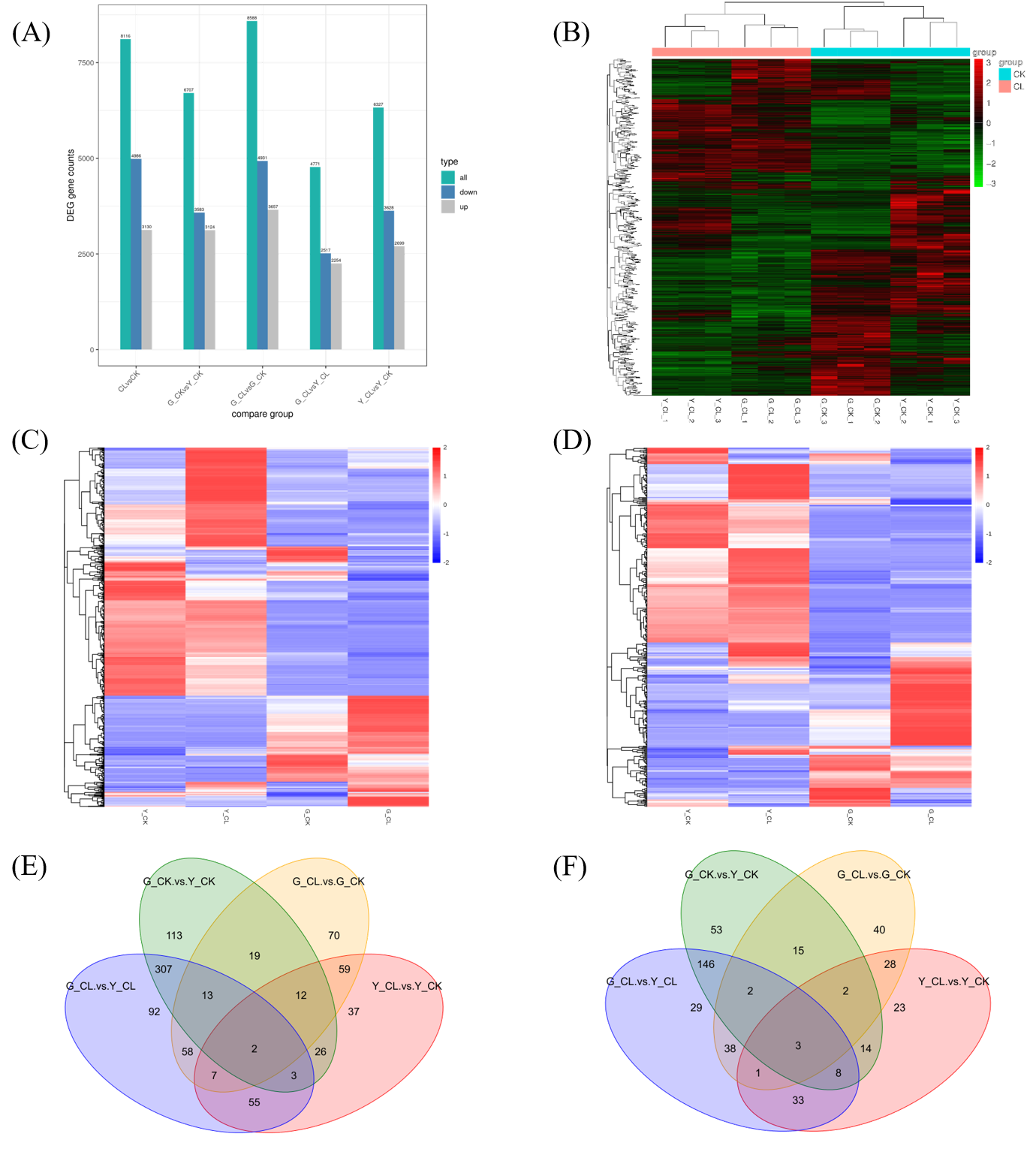


**Supplementary Figure S1.** Overview of Transcriptome sequencing analysis and widely targeted metabolome analysis. **(A)** The total number of differentially expressed genes (DEGs) and upregulated and downregulated DEGs under different treatments. **(B)** Heatmap visualization of the genes. The transition from green to red indicates a gradient of expression abundance for genes, ranging from low to high; the redder the color, the higher the expression abundance of differential metabolites. **(C)** (positive ion mode) and **(D)** (negative ion mode) Heatmap visualization of the differentially expressed metabolites (DEMs). The transition from blue to red indicates a gradient of expression abundance for metabolites, ranging from low to high; the redder the color, the higher the expression abundance of differential metabolites. **(E)** (positive ion mode) and **(F)** (negative ion mode) Venn diagram of DEMs.


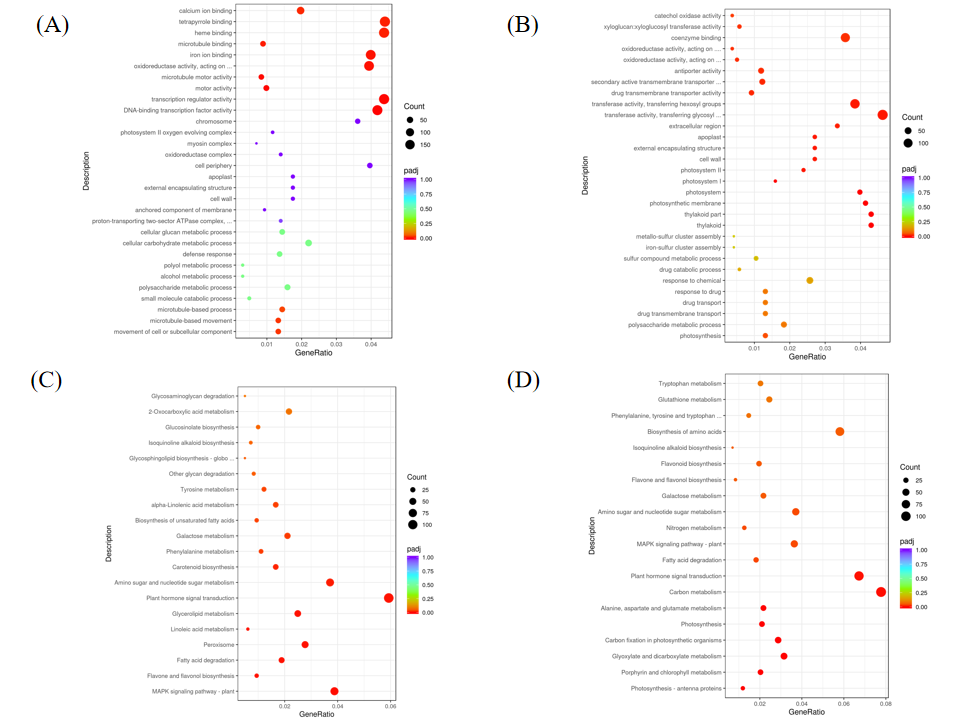


**Supplementary Figure S2.** The top 30 GO scatterplots and top 20 KEGG scatterplots. **(A)** GO terms of DEGs in G_CL vs G_CK. **(B)** GO terms of DEGs in Y_CL vs Y_CK. **(C)** KEGG pathway analysis of DEGs in G_CL vs G_CK. **(D)** KEGG pathway analysis of DEGs in Y_CL vs Y_CK.
