# Supplementary material for: Utilizing physiologies, transcriptomics, and metabolomics to unravel key genes and metabolites of Salvia miltiorrhiza Bge. seedlings in response to drought stress
Source: Front Plant Sci. 2025 Jan 14;15:1484688. doi: 10.3389/fpls.2024.1484688 (PMC11772496; doi:10.3389/fpls.2024.1484688)
Supplement: Supplementary file 2 [file Table2.doc]

| Sample | Raw_reads | Raw_bases | Clean_reads | Clean_bases | GC_pct | Exon | Intron | Intergenic |
| --- | --- | --- | --- | --- | --- | --- | --- | --- |
| G_CK_1 | 43350284 | 6.5G | 41390078 | 6.21G | 48.84 | 3993867020(74.6244%) | 228422467(4.2680%) | 1129671286(21.1076%) |
| G_CK_2 | 41014196 | 6.15G | 39161626 | 5.87G | 50.59 | 3888344618(76.2871%) | 140020529(2.7471%) | 1068620941(20.9657%) |
| G_CK_3 | 43047538 | 6.46G | 40875046 | 6.13G | 48.82 | 3974000112(75.1573%) | 209337204(3.9590%) | 1104236541(20.8836%) |
| G_CL_1 | 39635916 | 5.95G | 38102248 | 5.72G | 52.2 | 3192318868(68.6284%) | 121403838(2.6099%) | 1337876220(28.7616%) |
| G_CL_2 | 45280052 | 6.79G | 43923952 | 6.59G | 52.57 | 3273861931(68.0324%) | 124163819(2.5802%) | 1414186293(29.3874%) |
| G_CL_3 | 42413454 | 6.36G | 40652124 | 6.1G | 52.01 | 3210091466(67.5894%) | 137995308(2.9055%) | 1401314870(29.5051%) |
| Y_CK_1 | 39168292 | 5.88G | 38464340 | 5.77G | 50.06 | 3910295510(78.1626%) | 137754623(2.7536%) | 954722098(19.0839%) |
| Y_CK_2 | 44249682 | 6.64G | 42482050 | 6.37G | 53.55 | 4359944676(77.7646%) | 135832027(2.4227%) | 1110818052(19.8127%) |
| Y_CK_3 | 45758000 | 6.86G | 44537408 | 6.68G | 48.82 | 4224785794(75.1419%) | 187139708(3.3285%) | 1210483944(21.5296%) |
| Y_CL_1 | 42512266 | 6.38G | 40907184 | 6.14G | 52.27 | 4000032892(75.0151%) | 138326494(2.5941%) | 1193943617(22.3908%) |
| Y_CL_2 | 48311630 | 7.25G | 46516740 | 6.98G | 51.87 | 4590435908(75.8472%) | 156784423(2.5905%) | 1304997198(21.5623%) |
| Y_CL_3 | 41765066 | 6.26G | 40662032 | 6.1G | 53.15 | 3983072892(75.7319%) | 122161380(2.3227%) | 1154205827(21.9454%) |

Table S1. The sequence data for transcriptome profile.

***GC_pct**: GC content percentage; **Exon**: The number and percentage of clean data mapped to the exon regions of genome; **Intron**: The number and percentage of clean data mapped to the intron regions of genome; **Intergenic**: The number and percentage of clean data mapped to the intergenic regions of genome.
